# Impact of prevention in primary care on costs in primary and secondary care for people with serious mental illness

**DOI:** 10.1002/hec.4623

**Published:** 2022-10-30

**Authors:** Jemimah Ride, Panos Kasteridis, Nils Gutacker, Hugh Gravelle, Nigel Rice, Anne Mason, Maria Goddard, Tim Doran, Rowena Jacobs

**Affiliations:** ^1^ Health Economics Unit Melbourne School of Population and Global Health University of Melbourne Parkville Victoria Australia; ^2^ Centre for Health Economics University of York York UK; ^3^ Health Sciences University of York York UK

**Keywords:** data linkage, econometric analysis, healthcare costs, mental health, prevention

## Abstract

A largely unexplored part of the financial incentive for physicians to participate in preventive care is the degree to which they are the residual claimant from any resulting cost savings. We examine the impact of two preventive activities for people with serious mental illness (care plans and annual reviews of physical health) by English primary care practices on costs in these practices and in secondary care. Using panel two‐part models to analyze patient‐level data linked across primary and secondary care, we find that these preventive activities in the previous year are associated with cost reductions in the current quarter both in primary and secondary care. We estimate that there are large beneficial externalities for which the primary care physician is not the residual claimant: the cost savings in secondary care are 4.7 times larger than the cost savings in primary care. These activities are incentivized in the English National Health Service but the total financial incentives for primary care physicians to participate were considerably smaller than the total cost savings produced. This suggests that changes to the design of incentives to increase the marginal reward for conducting these preventive activities among patients with serious mental illness could have further increased welfare.

## 1 INTRODUCTION

Preventive practices in primary care can be an effective means of improving patient health outcomes in various domains of health and can reduce future healthcare expenditure (Cornuz et al., 2006; Garrett et al., 2011; Park et al., 2013). Participation in preventive activities by primary care physicians will depend on a number of behavioral factors, including their degree of altruism and intrinsic motivation (McGuire, 2000; Rebitzer & Taylor, 2011), their time preferences, the way in which they are paid (salary, capitation, fee for service, pay for performance) (Dahrouge et al., 2012; Iezzi et al., 2014; Town et al., 2005), and the impact of preventive activities on their remuneration. Preventive activities could increase costs in primary care even while improving patient health and reducing costs in other healthcare sectors (e.g., Gupta et al., 2019). An important—but largely unexplored—component of the incentive for primary care prevention is the extent to which the physician is the residual claimant for any cost savings resulting from their preventive activities (Meacock et al., 2014). The design of incentives and other measures to promote prevention by primary care physicians therefore requires information on the effect of prevention on primary care costs and on costs elsewhere in the healthcare system.

Previous research has investigated the association between the care incentivized under pay‐for‐performance schemes in primary care and changes in utilization in other healthcare settings, often without examining changes in primary care use or cost. Many of these have focused on care for chronic medical conditions. In the UK, several studies have investigated the impact of care incentivized through the quality and outcomes framework (QOF). Harrison et al. (2014) found moderate and sustained reductions in emergency hospital admissions for some ambulatory care sensitive conditions for which preventive care was incentivized under the QOF. Dusheiko et al. (2011) reported a negative association between achievement of QOF incentivized care (including, but not limited to preventive care) and hospital costs. Gupta et al. (2019) used a difference‐in‐differences approach to evaluate a Canadian pay‐for‐performance scheme that incentivized preventive primary care for patients with diabetes. They found higher primary care physician costs and lower diabetes‐related hospital costs for newly diagnosed patients but no difference in hospital costs for other conditions, even though the incentives targeted processes expected to affect comorbid conditions. Lavergne et al. (2016) examined the effect of incentive payments to Canadian primary care providers to encourage management of patients with complex conditions and found these were associated with higher costs overall, including increases in medical visits and hospital use. In the domain of mental health conditions, Ride et al. (2018, 2019) found that two QOF incentivized preventive activities in primary care—annual reviews and care plans—were associated with reduced utilization of hospital and specialist mental healthcare for patients with serious mental illness (schizophrenia, bipolar disorder, and other psychoses).

In this paper, we examine the impact of preventive primary care on costs for which the primary care physician is and is not the residual claimant in the context of care for patients with serious mental illness. Following Ride et al. (2018) we focus on two key services for patients with serious mental illness, namely care plans and annual reviews. We examine the association with primary care costs *and* on costs in other parts of the healthcare system, including specialist mental health services. These activities are incentivized under the QOF, but our analysis does not evaluate the QOF directly. Using a panel two‐part model to analyze patient‐level data linked across primary and secondary care sectors, we find that patients with care plans and annual reviews in the previous year have lower costs in the current quarter in primary care *and* in other healthcare sectors. We estimate that there are large beneficial externalities from these primary care preventive activities for patients with serious mental illness: the cost savings in other parts of the healthcare system from care plans and annual reviews in primary care are 4.7 times larger than the cost savings in primary care. Given that these savings outside primary care do not accrue to primary care providers, these findings may be important factors to consider when refining the incentive scheme for this patient group.

## 2 CONTEXT: PREVENTIVE PRIMARY CARE FOR SERIOUS MENTAL ILLNESS IN ENGLAND

The English National Health Service (NHS) operates a list system in which patients must register with a general practice that provides primary care services and acts as gatekeeper to secondary care. There were just over 8000 practices in 2012/2013, with an average list size of around 7000 patients (Prescribing and Primary Care Team, 2013). Most practices are small businesses that are owned and operated by general practitioner (GP) partnerships under contract with the NHS. Practices are paid by a mix of capitation per patient, quality incentive schemes, items of service, and lump sums (NHS Digital, 2019). Practices are reimbursed for the costs of their premises and information technology but fund all other expenses, such as salaried GPs, nurses, and clerical staff, from their revenue.

Capitation payments are determined by a national formula which takes account of the demographic mix of practice patients and local morbidity measures and contributes around 40% of practice revenue. Under the QOF, which is a national voluntary pay for performance scheme in England introduced in 2004, practices can earn up to 10% of their revenue by meeting a set of quality standards, many of which target preventive care (Moberly & Stahl‐Timmins, 2019; Roland, 2004). Practices receive incentive payments for clinical performance indicators linked to the proportions of patients with the relevant chronic condition for whom an indicator is achieved. No payment is made if the proportion is less than a lower threshold, and then increases linearly up an upper threshold of <100%. Practices are required to be able to provide information for post‐payment verification of their claims (NHS England, 2019). In prioritizing a prevention activity covered by the QOF, practices may consider its cost to them, the revenue received for meeting the QOF target, the improvement in the future health of patients, and the impact of prevention on future practice workload and costs. A key point for this analysis is that practices will not directly benefit from any reduction in future costs in other parts of the NHS that result from their prevention activities.

Serious mental illness imposes a high economic burden on health services (McCrone et al., 2008), including healthcare costs from hospitalization due to mental illness or physical comorbidities. This set of long term conditions are mainly managed in primary care, with the aim of preventing serious episodes which may then have to be treated by inpatient stays in specialist mental health units or by community mental health teams (Reilly et al., 2012). Under the QOF, practices are incentivized to document a patient‐specific care plan agreed with patients and caregivers for patients with serious mental illness, and to undertake an annual review of aspects of their physical health, including checks of blood pressure, body mass index, blood sugar, and cholesterol. Despite the QOF, patients with these conditions often receive less preventive care than the general population of patients (Lord et al., 2010; Solmi et al., 2020), a gap which has persisted despite the introduction of the QOF (Martin et al., 2014; Woodhead et al., 2016). In 2019/2020 only 71% of patients with serious mental illness received a care plan, 80% had a recording of blood pressure measurement, and 74% had body mass index recorded (NHS Digital, 2020) although at that time the upper threshold to achieve the maximum payment for each indicator was 90% (Primary Care Strategy and NHS Contracts Group, 2019). Understanding the effect of prevention on costs in primary care and elsewhere in the healthcare system may therefore be particularly pertinent for prevention policies targeting this patient population.

## 3 DATA

We use patient level data from 215 English practices included in the Clinical Practice Research Datalink (CPRD) GOLD, a large nationally representative database of primary care records, linked to national administrative datasets from hospitals and community mental health services. Data on all patients in the database with a diagnosis of serious mental illness (schizophrenia, bipolar disorder and other psychoses) and documented in primary care records from the start of their registration at each practice up to March 31, 2014 were included in the sample, provided that patient and practice records were of an acceptable standard for research as defined by a CPRD algorithm (Herrett et al., 2015), which includes valid age and gender, and recording of events in the clinical records. For costing, we used quarterly data from three financial years 2011/2012 to 2013/2014 (up to 12 quarters per patient). Financial years run from April 1 to March 31. For each patient with data from this period, we also obtained primary care records back to the beginning of their registration at the practice to allow us to include factors from their medical history in our analysis, including preventive activities in the 12 months prior to each quarter. We limited the sample to those for whom we had primary care records from 2010/2011 to capture care plans and annual reviews for 1 year prior to the start of the observation period. Further detail on the dataset including code lists used to identify diagnoses and items for costing is available in Ride et al. (2020).

Individual patient primary care records were linked to data from Hospital Episode Statistics (HES) and to community mental health service data from the Mental Health Services Dataset (MHSDS). HES has data on all general hospital admissions and presentations to emergency departments (ED) in public hospitals, including inpatient mental health admissions in general hospitals. MHSDS has data on care provided in publicly funded community mental health services and inpatient admissions in specialty mental health services. Outpatient specialist mental health services are included in our dataset, but outpatient services in acute hospitals such as clinic visits could not be included because of a limit on the number of datasets which could be linked.

For each patient we calculated quarterly costs within primary care and in three other sectors: general hospital inpatient admissions (divided into elective and unplanned admissions), ED presentations, and specialist mental health services. Costs were calculated using a bottom‐up approach from the perspective of the NHS, applying national average unit costs to instances of utilization from each data source. Primary care costs included visits to GPs and practice nurses, plus prescribed medications, and tests. Of these, only the costs of visits are borne by the practice. Medications and tests initiated by GPs are funded through other parts of the NHS and we therefore distinguish between the costs of primary care visits and other primary care costs.

General hospital inpatient costs included admissions for both physical and mental health problems, classified as elective and unplanned using the admission type field in the HES data. ED costs covered all types of presentations. Specialist mental health costs covered all inpatient admissions in mental health services plus care in community mental health services, including visits to mental health professionals (doctors, nurses, support workers, and a range of allied health professionals), treatment with electroconvulsive therapy, and multi‐disciplinary team reviews. Costs were allocated to 12 quarters, from April 1 to June 30, 2011 (Q1 of 2011/2012) through to January 1 to March 31, 2014 (Q4 of 2013/2014). Full details of the costing methodology are described in Ride et al. (2020).

We use two time‐varying measures of the prevention activities incentivized by the QOF. For each quarter we have an indicator for whether a care plan was documented in the patient's primary care records within the previous 12 months (and similarly for annual reviews). For example, the care plan indicator for Q1 2011/2012 (April 1 to June 30, 2011) is 1 if the patient had a care plan documented by their GP between April 1, 2010 and March 31, 2011 and is zero otherwise. The 12‐month time window reflects the period specified in the QOF for annual care plans and annual reviews.^1^ The costs of care plans and annual reviews, which are provided by primary care staff (GPs and practice nurses), are included in the primary care visit cost for the quarter in which they occur.

From primary care records we also obtain demographic and clinical characteristics of patients that are associated with healthcare need, utilization, and cost. These are measured at the later of the beginning of Q1 2011/2012 or 1 year after registration with the practice and include age, gender, ethnicity, socioeconomic disadvantage of their small area of residence, diagnostic information, and the comorbidities included in the Charlson comorbidity index (Charlson et al., 1987). We excluded 64 individuals from the analysis due to missing data on socioeconomic status. Clinical information on diagnostic type of serious mental illness and comorbidities was based on the clinical coding system used in primary care records in the UK (Read codes) (Chisholm, 1990). We also include straight line distances from the patient's practice to the nearest general hospital and to the nearest inpatient mental health facility to account for differences in access to secondary care services.

## 4 ECONOMETRIC SPECIFICATION

We estimate the association of changes over time in individuals' receipt of preventive care and their costs in primary care and in three other healthcare sectors: inpatient general hospitals, EDs, and specialist mental healthcare. We estimate two models for costs in primary care (for visits and for other primary care) and four models for secondary care costs (inpatient general hospital elective, inpatient general hospital unplanned, ED, specialist mental health). Since our cost data are highly skewed, with a long right tail and a large mass point at zero, we estimate two‐part panel data models (Jones, 2011; Jones et al., 2013). The first part estimates the probability of observing non‐zero costs using a probit model. The second part models the level of cost for individuals observed with positive costs using a generalized linear model (Blough & Ramsey, 2000) with a Gamma variance function and log link function. The variance and link functions were chosen on the basis of results from a modified Park's test (Manning & Mullahy, 2001) and the Pregibon link test (Pregibon, 1980).

The model specification is

(1) $$\mathrm{Pr}\left(y_{it}>0|{CP}_{it},{AR}_{it},D_{t},Z_{i},c_{i}\right)=\Phi \left(\theta_{1}{CP}_{it}+\theta_{2}{AR}_{it}+\delta Z_{i}+\rho_{t}D_{t}+c_{i}\right),$$

(2) $$E\left[y_{it}|y_{it}>0,{\mathrm{CP}}_{it},{AR}_{it},D_{t},Z_{i},m_{i}\right]=\exp\left(\alpha_{1}{\mathrm{CP}}_{it}+\alpha_{2}{\mathrm{AR}}_{it}+\lambda Z_{i}+\tau_{t}D_{t}+m_{i}\right),$$

where Φ() is the standard normal cumulative density function. $y_{it}$ is the cost for individual *i* (i=1,…,n) in quarter *t* (t=1,…,12) if the individual is observed in quarter *t*. *y*_*it*_ is assumed to follow a Gamma distribution conditional on covariates and the unobserved individual effect, $m_{i}$. *CP*_*it*_ and *AR*_*it*_ are binary indicators for having a care plan or annual review up to 1 year before quarter *t*. $Z_{i}$ is a vector of observed time‐invariant explanatory variables. *D*_*t*_ is a vector of quarter‐year dummy variables. $c_{i}$ and $m_{i}$ are unobserved time‐invariant individual effects.

We parameterize the unobserved individual effects as linear functions of the within‐individual means of the time‐varying regressors (Mundlak, 1978) as:

(3) $$c_{i}=\psi +\xi \bar{W_{i}}+a_{i},a_{i}∼N\left(0,\sigma_{a}\right)\;m_{i}=\omega +\pi \bar{W_{i}}+v_{i},v_{i}∼N\left(0,\sigma_{v}\right)$$

where $\bar{W_{i}}$ is a vector of the means of time‐varying variables ${\bar{\mathrm{CP}}}_{i},{\bar{\mathrm{AR}}}_{i}$, and ${\bar{X}}_{i}$. ${\bar{X}}_{i}$ (and the other patient *i* means) are the means over the quarters in which there is data on *i*: ${\bar{X}}_{i}=T_{i}^{-1}\sum_{t=1}^{12}{s_{it}X}_{it}$, where *s*_*it*_ is an indicator for patient *i* contributing data in quarter *t* and *T*_*i*_ is the number of quarters in which they do so. Since the panel is unbalanced, $\bar{W_{i}}$ also includes the mean of the quarter dummies for each individual i (Wooldridge, 2019).

We calculate the average partial effect on cost of having a care plan as follows. First, we estimate both the probability of incurring positive health care expenditures and the expected level of expenditure conditional on having positive expenditure by setting *CP*_*it*_ = 1 (with all other explanatories including ${\mathrm{AR}}_{it}$ at their actual values in quarter *t* for patient *i*). The two parts are multiplied for each observation (*i* and *t*) to obtain an expected cost. We then repeat for all *i* and *t* by setting *CP*_*it*_ = 0 $\left({\mathrm{AR}}_{it}=0\right)$ and again multiply the two parts. The difference between these, i.e., setting *CP*_*it*_ = 1 and *CP*_*it*_ = 0, is then averaged (over both individuals and time) to obtain the total average partial effect. More formally, for care plans,

(4) $${\mathrm{APE}}_{\mathrm{CP}}=\frac{1}{N}\sum_{i=1}^{n}\sum_{t=1}^{12}s_{it}\left[\left\{\Phi \left(\theta_{1}{\mathrm{CP}}_{it}\left(=1\right)+..\right)\exp\left(\alpha_{1}{\mathrm{CP}}_{it}\left(=1\right)+..\right)\right\}-\left\{\Phi \left(\theta_{1}{\mathrm{CP}}_{it}\left(=0\right)+..\right)\exp\left(\alpha_{1}{\mathrm{CP}}_{it}\left(=0\right)+..\right)\right\}\right]$$

where $N=\sum_{i}^{n}T_{i}$. The arguments (.) in parentheses contain the set of covariates together with the vector $\bar{W_{i}}$, omitting the zero mean unobserved $a_{i}$ and $v_{i}$. For example, $\left(\theta_{1}{\mathrm{CP}}_{it}\left(=1\right)+..\right)=\left({\theta_{0}+\psi +\theta }_{1}{\mathrm{CP}}_{it}\left(=1\right)+\theta_{2}{\mathrm{AR}}_{it}+\beta X_{it}+\delta Z_{i}+h_{t}D_{t}++\xi \bar{W_{i}}\right)$. We follow the analogous procedure when calculating average partial effects for annual reviews.

We estimate the partial effects of care plans and annual reviews for each category of cost using the results from the six separate models for primary care, inpatient hospital care, ED, and specialist mental health care. Because of the importance of the distinction between the effects of prevention on the costs of primary care visits which are borne by the practice and the effects on costs borne by the rest of the NHS, we sum the partial effects across other primary care, ED, inpatient general hospital elective, inpatient general hospital unplanned, and specialist mental health services to obtain the total impact of care plans and annual reviews on costs falling outside general practice. We do this rather than estimating a single model for costs outside general practice because of the differences in the proportions of zero cost observations in these sectors, the different effects of prevention in each sector, and the non‐linearity of the models.

We calculate bootstrapped standard errors clustered at practice level (Cameron et al., 2008) for the estimated partial effects using resampling with replacement from the original sample as

(5) $${se}_{\mathrm{APE}}=\sqrt{\frac{1}{J-1}\sum_{j=1}^{J}{\left({\mathrm{APE}}^{j}-\bar{\mathrm{APE}}\right)}^{2}}$$

where *J* = 1000 is the number of replications, ${\mathrm{APE}}^{j}$ is the estimated partial effect in replication *j*, and $\bar{\mathrm{APE}}$ is the mean of the bootstrapped estimates.

To explore heterogeneity in the association between care plans/annual reviews and healthcare costs, we also estimate each model including interactions between the preventive care indicators and older age (over 65 years), gender, and recency of diagnosis of serious mental illness.

## 5 RESULTS

The estimation sample had 150,748 person‐quarter observations from 16,485 patients, with a mean of 9.1 observations per individual, and 56% of the sample (9225 individuals) observed for the full 12 quarters of the study period. Table 1 provides patient summary statistics. Care plans (annual reviews) were observed in the prior 12 months for 26% (61%) of person‐quarter observations. The majority of these had the most recent care plan (79%) or annual review (82%) within the prior 3 months.

**TABLE 1:** Sample characteristics

Characteristic	*N* (patients)	%
Age (years)		
19–35	3410	20.7
36–45	3390	20.6
46–55	3397	20.6
56–65	2616	15.9
66+	3672	22.3
Sex		
Female	8313	50.4
Male	8172	49.6
Ethnicity		
Black or minority ethnicity	4690	28.5
White	11,795	71.5
Index of multiple disadvantage quintile		
1 (least disadvantaged)	2630	16.0
2	3033	18.4
3	3038	18.4
4	3825	23.2
5 (most disadvantaged)	3959	24.0
Diagnostic category		
Schizophrenia	8575	52.0
Bipolar disorder	6076	36.9
Both diagnostic categories	1834	11.1
Years since diagnosis at start of observation period		
0–1	3228	19.6
2–5	3148	19.1
>5	10,109	61.3
Comorbidities		
Depression	10,138	61.5
Diabetes	1299	7.9
Cancer	700	4.2
Cerebrovascular disease	542	3.3
Chronic pulmonary disease	2902	17.6
Coronary heart disease or myocardial infarction	372	2.3
Dementia	300	1.8
Peptic ulcer	352	2.1
Peripheral vascular disease	161	1.0
Chronic kidney disease	934	5.7
Rheumatological disease	291	1.8
Liver disease	97	0.6
Smoking		
Current or past	12,667	76.8
Non‐smoker	3818	23.3

Quality indicator	*N* (periods)	%
Care plan		
Present	39,656	26.3
Annual review		
Present	92,364	61.3

*Note*: Based on 16,485 patients with serious mental illness and 150,748 periods in the estimation sample.

The costs incurred in primary care and the other sectors are summarized in Table 2. Most health care costs accrued in sectors outside primary care, with £235 (17% of the total quarterly cost per patient) incurred in primary care, comprised of £59 in primary care visits whose costs fall on the practice and £176 in other resource use initiated by GPs (tests and medications) but not borne by the practice. Costs in elective and unplanned inpatient general hospital admissions, ED, and specialist mental health care totaled £1368. Zero quarterly costs were common in specialist mental health (63%) and especially in hospital costs—ED 91%, elective admissions 96%, and unplanned admissions 94% of quarterly observations.

**TABLE 2:** Costs per patient with serious mental illness per quarter

	Health care costs in full sample		Patient‐periods with zero costs	Health care costs in periods with non‐zero costs	
	Mean	SD	%	Mean	SD
Primary care visits	58.99	93.78	35.62	91.63	103.29
Other primary care	176.08	333.30	14.48	205.89	351.79
Elective admissions	168.01	1445.79	95.73	3935.17	5843.02
Unplanned admissions	304.08	1779.97	93.88	4969.09	5347.48
Emergency department	16.67	68.24	90.79	180.96	144.34
Specialist mental health care (inpatient and community)	643.86	3317.43	62.77	1729.64	5261.81

*Note*: Costs per patient with serious mental illness per quarter are measured in 2013/2014 GBP (2014 £1 equivalent to 2014 US $1.43 and 2019 US $1.08).

The estimated coefficients for care plans and annual reviews from the two‐part models for each sector of costs are presented in Table 3. Having a care plan documented in the previous 12 months was associated with a lower probability of incurring costs in the current quarter for primary care visits and unplanned hospital inpatient care, but not in other primary care costs (tests and medication), elective hospital inpatient care, or ED or specialist mental health care. For those with non‐zero costs in the quarter, care plans were associated with lower costs of primary care visits and specialist mental health care.

**TABLE 3:** Regression coefficients on primary care prevention indicators from two‐part model for costs per quarter

	Primary care visits		Other primary care		Inpatient general hospital elective		Inpatient general hospital unplanned		Emergency department		Specialist mental health	
	Pr positive cost	Cost if positive	Pr positive cost	Cost if positive	Pr positive cost	Cost if positive	Pr positive cost	Cost if positive	Pr positive cost	Cost if positive	Pr positive cost	Cost if positive
	*β* (SE)	*β* (SE)	*β* (SE)	*β* (SE)	*β* (SE)	*β* (SE)	*β* (SE)	*β* (SE)	*β* (SE)	*β* (SE)	*β* (SE)	*β* (SE)
Main model												
Care plan	−0.15*** (0.01)	−0.08*** (0.01)	−0.04 (0.02)	−0.01 (0.01)	−0.01 (0.02)	0.003 (0.03)	−0.07** (0.02)	−0.03 (0.03)	0.02 (0.02)	0.00 (0.02)	0.00 (0.03)	−0.11*** (0.02)
Annual review	−0.12*** (0.01)	−0.06*** (0.01)	0.03 (0.02)	−0.02** (0.006)	0.03 (0.02)	−0.05 (0.03)	−0.05* (0.02)	0.03 (0.03)	−0.03* (0.02)	0.03 (0.01)	−0.03 (0.02)	−0.10*** (0.02)
Model including interaction of preventive care indicators with patient characteristics												
Care plan	−0.10*** (0.02)	−0.07*** (0.01)	0.02 (0.04)	−0.001 (0.01)	−0.005 (0.04)	−0.01 (0.04)	−0.11** (0.03)	−0.05 (0.04)	0.007 (0.03)	−0.01 (0.02)	0.01 (0.03)	−0.08* (0.03)
X age over 65	−0.03 (0.03)	−0.01 (0.02)	−0.09 (0.08)	−0.01 (0.01)	0.10* (0.05)	0.01 (0.05)	0.11** (0.04)	0.05 (0.05)	0.03 (0.03)	0.03 (0.03)	0.05 (0.05)	−0.03 (0.05)
X male	−0.05** (0.02)	−0.01 (0.02)	−0.05 (0.05)	−0.01 (0.01)	−0.09* (0.04)	0.08 (0.05)	0.08* (0.04)	0.05 (0.06)	0.01 (0.03)	0.02 (0.02)	0.03 (0.04)	−0.002 (0.04)
X recent diagnosis	−0.09* (0.03)	−0.03 (0.02)	−0.08 (0.06)	−0.01 (0.02)	0.04 (0.05)	−0.14* (0.06)	−0.12* (0.05)	−0.04 (0.05)	−0.02 (0.04)	0.002 (0.03)	−0.19*** (0.05)	−0.12** (0.05)
Annual review	−0.12*** (0.02)	−0.05** (0.01)	0.03 (0.03)	−0.005 (0.01)	0.06 (0.03)	0.01 (0.04)	−0.04 (0.03)	0.03 (0.04)	0.001 (0.02)	0.04* (0.02)	−0.01 (0.03)	−0.06* (0.03)
X age over 65	−0.06* (0.03)	−0.01 (0.02)	0.01 (0.06)	−0.01 (0.01)	0.01 (0.04)	−0.08 (0.06)	−0.05 (0.04)	−0.04 (0.05)	−0.07* (0.03)	−0.06* (0.02)	−0.02 (0.05)	−0.10 (0.04)
X male	0.07** (0.02)	−0.01 (0.01)	0.05 (0.04)	−0.01 (0.01)	−0.001 (0.04)	−0.10* (0.05)	0.03 (0.03)	0.04 (0.05)	−0.02 (0.03)	−0.006 (0.02)	0.02 (0.04)	−0.004 (0.04)
X recent diagnosis	−0.14*** (0.03)	−0.05* (0.02)	−0.22*** (0.06)	−0.06** (0.02)	−0.19*** (0.05)	0.07 (0.07)	−0.06 (0.04)	0.002 (0.06)	−0.05 (0.03)	−0.005 (0.03)	−0.11* (0.04)	−0.11* (0.05)

*Note*: Standard errors adjusted for clustering at the practice level. Results from two‐part models: first part random effects probit and second part random effects glm second part (log link and gamma family) including quarter dummies and means of time‐varying characteristics (care plan and annual review, and as the panel is unbalanced also quarter dummies). Full main model results available in Appendix. Recent diagnosis: serious mental illness diagnosed within 2 years of baseline.

*p < 0.05, **p < 0.01, ***p < 0.001.

The estimated average partial effects reported in Table 4 indicate that care plans are associated with lower costs of primary care visits, unplanned inpatient hospital care, and specialist mental health care. The reduction in costs is larger for costs borne outside primary care practices (£34.90, 95% CI £19.45–£50.35) than those incurred by primary care practices (£7.41, 95% CI £6.39–£8.43), although it is a smaller proportionate reduction compared to the mean quarterly costs in each sector (2.7% vs. 12.6%).

**TABLE 4:** Average partial effects of care plans and annual reviews on costs

Cost sector	Care plans		Annual reviews	
	£/quarter (95% CI)	Change as % mean	£/quarter (95% CI)	Change as % mean
Costs based in primary care practices				
Primary care visits	−7.41*** (−8.43, −6.39)	−12.6%	−6.05*** (−7.11, −5.00)	−10.3%
Costs based outside primary care practices				
Other primary care	−1.04 (−2.29, 0.21)	−0.6%	−1.81** (−3.14, −0.48)	−1.0%
Inpatient general hospital elective	−1.06 (−5.79, 3.66)	−0.3%	1.07 (−3.30, 5.44)	0.3%
Inpatient general hospital unplanned	−17.68** (−27.96, −7.38)	−10.5%	−8.56 (−19.22, 2.09)	−5.1%
Emergency department	0.34 (−0.34, 1.01)	2.0%	−0.37 (−1.00, 0.25)	−2.2%
Specialist mental health care (inpatient and community)	−15.45*** (−22.07, −8.83)	−2.4%	−18.80*** (−26.49, −11.12)	−2.9%
Total costs based outside primary care practices^a^	−34.90*** (−50.35, −19.45)	−2.7%	−28.48** (−44.91, −12.06)	−2.2%

*Note*: Bootstrapped standard errors are adjusted for clustering at the practice level. The partial effect for non‐primary care is calculated as the sum of the partial effects of three non‐primary care components, with standard errors obtained by bootstrapping. Estimates are from the model without interactions.

Abbreviation: ED, emergency department.

^a^ Includes non‐visit primary care (tests, medication), ED, inpatient general hospital elective and unplanned, and specialist mental health care.

*p < 0.05, **p < 0.01, ***p < 0.001.

Annual reviews were associated with a lower probability of positive cost for primary care visits, unplanned hospital inpatient care, and ED attendances, and conditional on incurring costs, they were associated with lower costs in primary care visits, other primary care, and specialist mental health care. Overall, as shown in Table 4, annual reviews were associated with lower primary care visit costs, lower other primary care costs, and lower specialist mental health costs. As with care plans, the reduction in costs associated with annual reviews is larger for costs based outside primary care practices (£28.48, 95% CI £12.06–£44.91) than based in primary care practices (£6.05, 95% CI £5.00–£7.11). The full two‐part model results for each sector are available in Appendix.

There is evidence of heterogeneity in the effect of care plans and annual reviews across some groups of patients, as seen in the results in the second panel of Table 3. Annual reviews are associated with lower probability of costs and/or lower level of costs for patients whose serious mental illness was diagnosed more recently^2^ in all downstream cost sectors except ED care and unplanned hospital admissions. Care plans are also associated with lower specialist mental health costs, lower level of elective admission costs, and lower probability of unplanned admission costs for this patient group. Annual reviews are associated with lower level of ED costs for patients over 65 and lower elective admission costs for males. The association of care plans with lower unplanned admission costs is not present in older patients, who also have a positive association between care plans and elective admission costs. Care plans in males are associated with lower probability of elective admission costs but for unplanned admissions the lower probability is smaller than for females.

To understand whether patients who contribute more to savings in secondary care also contribute a larger share of savings in primary care, we calculated the Pearson's product‐moment correlation between the estimated total savings held outside primary care and the savings held in primary care for each care indicator. There was a moderate positive correlation for both care plans, *r*(16,483) = 0.449, *p* < 0.001, and for annual reviews, *r*(16,483) = 0.476, *p* < 0.001.

## 6 DISCUSSION

Many healthcare systems seek to shift the emphasis from curative care and toward disease prevention to improve population health and reduce overall expenditure. These efforts may be hampered if the providers tasked with delivering preventive care—usually in primary care—are not sufficiently incentivized to engage in these activities because, whilst they bear the cost of prevention, they do not benefit from any resulting cost savings in other sectors.

We examined this issue using two preventive primary care activities targeted at patients with long term serious mental illness in the English NHS. We estimated panel two‐part models accounting for time‐invariant unobserved patient characteristics to examine the impact of care plans and annual physical reviews on costs in primary care, elective and unplanned general hospital admissions, ED, and specialist mental health. We found that if a patient with serious mental illness had these types of preventive care delivered in the previous 12 months their costs to the primary care practice in the current quarter were lower by 12.6% with a care plan and by 10.3% with an annual review. We also found that if the patient had a care plan in the previous 12 months, their unplanned hospital admission costs in the current quarter were 10.5% lower.

The annual reduction in primary care practice cost due to each care plan was £29.64 and from an annual review was £24.20. With an average of 44 care plans and 42 annual reviews per practice in 2012/2013, the annual reduction in per practice costs from these preventive activities were £1307 and £1017 respectively. These primary care cost reductions could provide GPs with a financial incentive for care plans and annual reviews, in addition to reimbursement received through the QOF (an average of £801 for care plans and £3070 for annual reviews in 2012/2013.^3^). This incentive would be partly offset by the associated clinical and administrative costs, which we do not observe. Although broad estimates of GP expenses are published (e.g., Curtis & Burns, 2015) the costs attributable to QOF indicators are unknown.

The care plans and annual reviews in an average general practice generated total cost reductions across primary care, general hospitals, EDs, and specialist mental health providers of £7464 from care plans and £5804 from annual reviews. The financial gain (cost saving in the practice plus QOF payment) to the average practice from care plans was 28% ([£1307 + £801]/£7464) of the total cost reduction across all health care sectors from care plans. For annual reviews the financial gain to the average practice was a much larger 70% ([£1017 + £3070]/£5804) of the total cost reduction, mainly because of the larger QOF payment for annual reviews.

There is a substantial body of literature evaluating various aspects of the impact of the QOF, including process outcomes (such as quality of care, care coordination), health outcomes (such as mortality or hospital admissions for ambulatory care sensitive conditions), inequalities in care or health outcomes, and costs (including Boeckxstaens et al., 2011; Forbes et al., 2017; Gillam et al., 2012; Grigoroglou et al., 2020; Walker et al., 2010). These studies have found mixed effects of the QOF, with concerns that impacts on processes of care have limited translation into improved health outcomes. Other studies have examined aspects of the QOF and changes to the QOF on GP behavior and functioning of primary care (e.g., Allen et al., 2018; Gilbert et al., 2019; Wilding et al., 2018). In contrast, our analysis has not evaluated the impact of the QOF per se, nor does it seek to estimate the impact of changes to QOF design. Rather, we evaluate the impact of the incentivized activities on costs across different healthcare sectors, with a view to informing the design of the QOF. To the best of our knowledge, no other studies have examined this issue, being focused on either evaluating the QOF or other P4P schemes themselves, or not including costs across both primary and secondary care.

Our analysis is able to address gaps in previous literature by examining the impact of primary care prevention on costs in both primary and secondary care, including general hospital inpatient and ED care and specialist community and hospital‐based mental health services. However, our dataset did not include outpatient care provided by acute general hospitals, such as outpatient clinic visits. If costs of outpatient hospital visits increased, for example, due to increased preventive referrals by the general practice, our results will overestimate cost savings in secondary care.

We analyze GP activities but do not account for patient engagement or adherence with the recommendations and treatment plan arising from a care plan or annual review. Its impact on our results would depend on the number and characteristics of non‐adherent patients, and on GPs' responses in their treatment of those patients. If some patients in our dataset were disengaged from the care plans/annual reviews conducted by GPs, then our results might underestimate the potential impact of the preventive care that would have resulted if they were engaged. However, if lack of patient engagement led to the GP not undertaking preventive care with certain patients, then our estimates would overestimate the potential benefit of extending this type of care to the patients who missed out, since they may be less likely to comply.

Some GPs may be more expert at the management of patients with serious mental illness, which may be reflected in the quality of care plans or annual reviews they conduct as well as the rest of their care of the patient. We do not observe the quality of these care plans/annual reviews and so our analysis estimates the average difference in healthcare costs associated with each. While the care plans or annual reviews conducted by more expert GPs might have more impact on reducing downstream costs, these GPs may (appropriately) conduct more preventive and comprehensive care, which may offset more of the savings. If expert GPs are more likely to conduct care plans or annual reviews, then increasing the rate of care plans or annual reviews among less expert GPs through incentives in the QOF may result in higher or lower cost differences than we those estimate.

Our analytical approach accounts for time‐invariant patient‐level fixed effects, using longitudinal variation within individuals' care. It is possible that other forms of care provided by GPs such as changes to medication may be conducted in tandem with care plans and/or annual reviews, which could influence our results if those other forms of care had an impact on healthcare costs in the following year. However, this reflects the rationale of incentivizing these activities, that is to promote comprehensive management of the patient's needs.

Any financial gain to the practices could be a direct incentive to partners in the practice, but the preventive activities could be conducted by staff who are not partners (e.g., salaried GPs or practice nurses). These staff members could receive an incentive from prevention of future workload but may not receive a financial incentive unless practice‐level structures were in place to incentivize the activities.

The study data were taken from patients who attended general practices participating in CPRD data linkages. The CPRD set of practices is broadly nationally representative in terms of the age–sex composition and socio‐economic profile of their patient population. However, our sample may be less representative of the national population of people with serious mental illness.

### 6.1 Policy implications

The total financial incentive to practices (the reduction in practice costs and QOF payments) was considerably smaller than the total cost savings identified from care plans and annual reviews. Under the funding arrangements for primary care in the UK, savings that return to the GP practice do not benefit the national funder but can incentivize GP performance. Our results show that these incentives are small in comparison to the magnitude of savings elsewhere in the NHS. Our findings suggest that changes to the design of QOF payments in the domain of serious mental illness, especially increases in the marginal reward for additional care plans and annual reviews, could increase welfare if they led to additional increases in preventive primary care and if these then generated further savings in secondary care. The assessment of such potential changes to the QOF would need to consider that cost savings in secondary care are partly offset by the QOF payments to GPs to incentivize these activities.

Such redesign of the QOF would also require detailed estimates of how practices would respond to changes in QOF payment for these types of prevention. Under the QOF, increasing the proportion of patients receiving the specified care above a threshold does not increase payments. This threshold was 55% for care plans for patients with serious mental illness, but 81% had a care plan in 2012/2013 (the period in which this analysis was conducted) (NHS Digital, 2013), suggesting that practices were motivated by a combination of financial and non‐financial considerations, such as reputational rewards and patient benefit (Allen et al., 2018). Rates of achievement have decreased over time and as noted earlier, in 2019/2020 the proportion of patients with serious mental illness who had received a care plan had dropped to 71% even though the upper threshold had increased to 90% (NHS Digital, 2020), suggesting that there is room to further incentivize primary care to provide these types of care.

The larger cost savings for patients with a recent diagnosis of serious mental illness may be of particular interest to policymakers, since this group had higher costs overall than patients diagnosed more than 2 years prior. These larger savings were seen in the most expensive categories of cost, inpatient hospital care, and specialist mental health care.

The finding of a positive correlation between savings held by primary care practices and wider savings in secondary care suggests that if care plans and annual reviews prevent avoidable GP care they are also likely to prevent avoidable use of secondary care, consistent with the common goal of patient benefit across sectors for GPs, secondary care, and policymakers.

## 7 CONCLUSIONS

This analysis of linked primary and secondary healthcare data for patients in England with serious mental illness has shown that two types of preventive activity in primary care (care plans and annual reviews of physical health) are associated with large beneficial externalities for which the primary care physician is not the residual claimant. These activities are incentivized under the national primary care incentive scheme, the QOF, but the total financial incentives for primary care physicians to conduct these activities were considerably smaller than the total cost savings produced. Given the falling rates of achievement in the incentives scheme, changes to the design of the incentives scheme to increase incentives for primary care physicians could further increase welfare.

## CONFLICT OF INTEREST

The author declares that there is no conflict of interest that could be perceived as prejudicing the impartiality of the research reported.

## Supporting information

Supporting Information S1

## Data Availability

The data that support the findings of this study are available from CPRD. Restrictions apply to the availability of these data, which were used on application to CPRD for this study. Data are not able to be shared by the authors.

## ENDNOTES

^1^
The care plan definition in QOF was unchanged over the period of this study. The QOF definition of an annual review changed in 2011/2012 from the patient being given health promotion and prevention advice to more specific indicators (including blood pressure, body mass index, cholesterol, glucose). The annual review indicator takes the value 1 if the patient had any three of these four checks recorded within a 3‐month period. There is no formal definition of the time and effort required to complete a care plan or annual review (NHS Employers, NHS Commissioning Board, & General Practitioners Committee, 2013) but clinical advice suggests these could be completed in a single visit.

^2^
Within 2 years prior to the start of the observation period.

^3^
353,695 care plans were documented across all practices in England in 2012/2013, with 47,965 QOF points awarded to practices. The national average price per point was £134, and 8020 practices took part in the QOF (Prescribing and Primary Care Team, 2013), an average of £801 per practice for care plans. There were 336,988 annual reviews yielding 183,748 QOF points in the same period, with the average practice paid £3070 for annual reviews.
