# Supplementary material for: Impact of prevention in primary care on costs in primary and secondary care for people with serious mental illness
Source: Health Econ. 2022 Oct 30;32(2):343–55. doi: 10.1002/hec.4623 (PMC10092448; doi:10.1002/hec.4623)
Supplement: Supplementary file 1 — Supporting Information S1 [file HEC-32-343-s001.docx]

**Supplementary Table 1.** **Regression results from two-part model for primary care visit costs**

|  | Pr positive cost | | Cost if positive | |
| --- | --- | --- | --- | --- |
|  | β (se) | | β (se) | |
| **Primary care quality indicators** |  |  |  |  |
| Care plan | -0.15*** | (0.01) | -0.08*** | (0.01) |
| Annual review | -0.12*** | (0.01) | -0.06*** | (0.01) |
| **Patient characteristics** |  |  |  |  |
| Age at 1 April 2011 *base: 18-35 years* |  |  |  |  |
| 36-45 | 0.02 | (0.02) | 0.03 | (0.02) |
| 46-55 | 0.05* | (0.02) | 0.04* | (0.02) |
| 56-65 | 0.08* | (0.03) | 0.05* | (0.02) |
| 66+ | 0.16*** | (0.03) | 0.01 | (0.02) |
| Gender *base: female* |  |  |  |  |
| Male | -0.32*** | (0.02) | -0.13*** | (0.01) |
| Ethnicity *base: Black and minority ethnicities* |  |  |  |  |
| White | 0.16*** | (0.03) | 0.10*** | (0.02) |
| Index of Multiple Deprivation for area of residence *base: 1 (least disadvantaged)* |  |  |  |  |
| 2 | -0.06 | (0.03) | 0.002 | (0.02) |
| 3 | -0.13*** | (0.03) | -0.03 | (0.03) |
| 4 | -0.10** | (0.04) | -0.06 | (0.03) |
| 5 (most disadvantaged) | -0.14** | (0.04) | -0.13** | (0.04) |
| Diagnosis category *base: bipolar* |  |  |  |  |
| Schizophrenia | -0.13*** | (0.02) | -0.08*** | (0.01) |
| Both diagnoses | -0.06* | (0.03) | -0.06* | (0.01) |
| Number of years since diagnosis *base: 0-1* |  |  |  |  |
| 2-5 | -0.16*** | (0.03) | -0.08*** | (0.02) |
| 5+ | -0.15*** | (0.03) | -0.09*** | (0.02) |
| Comorbidities *base: each not present* |  |  |  |  |
| Depression | 0.19*** | (0.02) | 0.15*** | (0.01) |
| Diabetes | 0.25*** | (0.03) | 0.11*** | (0.02) |
| Chronic pulmonary disease | 0.25*** | (0.02) | 0.16*** | (0.01) |
| Cerebrovascular disease | 0.10* | (0.05) | 0.01 | (0.04) |
| CHD or myocardial infarction | 0.12* | (0.05) | 0.10** | (0.04) |
| Dementia | 0.005 | (0.07) | -0.34*** | (0.05) |
| Cancer | 0.14*** | (0.04) | 0.12*** | (0.03) |
| Peptic ulcer disease | 0.20*** | (0.05) | 0.07 | (0.04) |
| Peripheral vascular disease | 0.17* | (0.08) | 0.14* | (0.06) |
| Chronic kidney disease | 0.18*** | (0.03) | 0.04 | (0.03) |
| Rheumatological disease | 0.22*** | (0.06) | 0.19*** | (0.04) |
| Liver disease | 0.26* | (0.11) | 0.15* | (0.06) |
| Smoking *base: non-smoker* |  |  |  |  |
| Current or ex-smoker | 0.09*** | (0.02) | 0.08*** | (0.01) |
| **Practice characteristics** |  |  |  |  |
| Distance from practice to nearest general hospital *base 0-3km* |  |  |  |  |
| 3-6km | -0.10* | (0.05) | -0.05 | (0.04) |
| 6-9km | 0.06 | (0.06) | -0.02 | (0.05) |
| >9km | -0.05 | (0.07) | -0.002 | (0.04) |
| Distance from practice to nearest mental health inpatient facility *base 0-3km* |  |  |  |  |
| 3-6km | 0.04 | (0.06) | -0.03 | (0.05) |
| 6-9km | -0.05 | (0.06) | -0.10 | (0.06) |
| >9km | 0.02 | (0.06) | -0.06 | (0.04) |
| N observations | 150,748 |  | 97,056 |  |
| N individuals | 16,485 |  | 15,462 |  |

Note: *p<0.05 **p<0.01 ***p<0.001. Standard errors adjusted for clustering at the practice level. Results from two part models: random effects probit and random effects glm second part (log link and gamma family) including quarter dummies and means of time-varying characteristics ((AR, CP, and, as the panel is unbalanced, quarter dummies)

**Supplementary Table 2.** **Regression results from two-part model for non-visit primary care costs**

|  | Pr positive cost | | Cost if positive | |
| --- | --- | --- | --- | --- |
|  | β (se) | | β (se) | |
| **Primary care quality indicators** |  |  |  |  |
| Care plan | -0.04 | (0.02) | -0.01 | (0.01) |
| Annual review | 0.03 | (0.02) | -0.02** | (0.01) |
| **Patient characteristics** |  |  |  |  |
| Age at 1 April 2011 *base: 18-35 years* |  |  |  |  |
| 36-45 | 0.29*** | (0.05) | 0.27*** | (0.03) |
| 46-55 | 0.57*** | (0.06) | 0.42*** | (0.03) |
| 56-65 | 0.87*** | (0.07) | 0.58*** | (0.04) |
| 66+ | 1.43*** | (0.08) | 0.71*** | (0.04) |
| Gender *base: female* |  |  |  |  |
| Male | -0.55*** | (0.04) | -0.16*** | (0.02) |
| Ethnicity *base: Black and minority ethnicities* |  |  |  |  |
| White | 0.40*** | (0.04) | 0.36*** | (0.02) |
| Index of Multiple Deprivation for area of residence *base: 1 (least disadvantaged)* |  |  |  |  |
| 2 | 0.02 | (0.07) | 0.03 | (0.03) |
| 3 | -0.05 | (0.08) | 0.09* | (0.04) |
| 4 | 0.06 | (0.08) | 0.17*** | (0.04) |
| 5 (most disadvantaged) | 0.11 | (0.08) | 0.25*** | (0.04) |
| Diagnosis category *base: bipolar* |  |  |  |  |
| Schizophrenia | -0.40*** | (0.04) | -0.06** | (0.02) |
| Both diagnoses | -0.18** | (0.06) | 0.05 | (0.03) |
| Number of years since diagnosis *base: 0-1* |  |  |  |  |
| 2-5 | -0.14* | (0.06) | 0.01 | (0.03) |
| 5+ | -0.19** | (0.06) | -0.03 | (0.03) |
| Comorbidities *base: each not present* |  |  |  |  |
| Depression | 0.35*** | (0.04) | 0.18*** | (0.02) |
| Diabetes | 0.83*** | (0.09) | 0.64*** | (0.03) |
| Chronic pulmonary disease | 0.39*** | (0.05) | 0.40*** | (0.02) |
| Cerebrovascular disease | 1.10*** | (0.19) | 0.37*** | (0.05) |
| CHD or myocardial infarction | 1.01*** | (0.20) | 0.20*** | (0.05) |
| Dementia | 0.81** | (0.23) | 0.22*** | (0.06) |
| Cancer | 0.36** | (0.14) | 0.17*** | (0.04) |
| Peptic ulcer disease | 0.21 | (0.14) | 0.16** | (0.05) |
| Peripheral vascular disease | 0.32 | (0.29) | 0.24** | (0.07) |
| Chronic kidney disease | 1.01*** | (0.16) | 0.25*** | (0.03) |
| Rheumatological disease | 0.29 | (0.06) | 0.41*** | (0.07) |
| Liver disease | 1.12** | (0.37) | 0.48*** | (0.11) |
| Smoking *base: non-smoker* |  |  |  |  |
| Current or ex-smoker | 0.15*** | (0.04) | 0.10*** | (0.02) |
| **Practice characteristics** |  |  |  |  |
| Distance from practice to nearest general hospital *base 0-3km* |  |  |  |  |
| 3-6km | 0.07 | (0.08) | -0.01 | (0.04) |
| 6-9km | 0.08 | (0.10) | 0.04 | (0.05) |
| >9km | 0.17 | (0.09) | 0.04 | (0.06) |
| Distance from practice to nearest mental health inpatient facility *base 0-3km* |  |  |  |  |
| 3-6km | 0.04 | (0.10) | 0.03 | (0.06) |
| 6-9km | -0.03 | (0.10) | 0.01 | (0.06) |
| >9km | 0.23* | (0.09) | 0.13* | (0.06) |
| N observations | 150,748 |  | 128,922 |  |
| N individuals | 16,485 |  | 15,578 |  |

Note: *p<0.05 **p<0.01 ***p<0.001. Standard errors adjusted for clustering at the practice level. Results from two part models: random effects probit and random effects glm second part (log link and gamma family) including quarter dummies and means of time-varying characteristics ((AR, CP, and, as the panel is unbalanced, quarter dummies)

**Supplementary Table 3.** **Regression results from two-part model for general hospital elective inpatient costs**

|  | Pr positive cost | | Cost if positive | |
| --- | --- | --- | --- | --- |
|  | β (se) | | β (se) | |
| **Primary care quality indicators** |  |  |  |  |
| Care plan | -0.01 | (0.02) | 0.003 | (0.03) |
| Annual review | 0.03 | (0.02) | -0.05 | (0.03) |
| **Patient characteristics** |  |  |  |  |
| Age at 1 April 2011 *base: 18-35 years* |  |  |  |  |
| 36-45 | 0.05 | (0.04) | -0.06 | (0.05) |
| 46-55 | 0.10* | (0.03) | -0.02 | (0.05) |
| 56-65 | 0.25*** | (0.04) | -0.02 | (0.05) |
| 66+ | 0.20*** | (0.04) | 0.07 | (0.05) |
| Gender *base: female* |  |  |  |  |
| Male | -0.05* | (0.02) | -0.03 | (0.03) |
| Ethnicity *base: Black and minority ethnicities* |  |  |  |  |
| White | 0.44*** | (0.04) | -0.03 | (0.04) |
| Index of Multiple Deprivation for area of residence *base: 1 (least disadvantaged)* |  |  |  |  |
| 2 | 0.01 | (0.04) | 0.06 | (0.05) |
| 3 | 0.06 | (0.04) | 0.05 | (0.05) |
| 4 | 0.02 | (0.04) | 0.05 | (0.05) |
| 5 (most disadvantaged) | 0.01 | (0.04) | -0.03 | (0.05) |
| Diagnosis category *base: bipolar* |  |  |  |  |
| Schizophrenia | -0.09*** | (0.02) | 0.05 | (0.03) |
| Both diagnoses | 0.004 | (0.04) | -0.01 | (0.03) |
| Number of years since diagnosis *base: 0-1* |  |  |  |  |
| 2-5 | -0.07 | (0.04) | 0.05 | (0.05) |
| 5+ | -0.10** | (0.03) | 0.09* | (0.04) |
| Comorbidities *base: each not present* |  |  |  |  |
| Depression | 0.04 | (0.02) | -0.09** | (0.03) |
| Diabetes | 0.11** | (0.04) | 0.04 | (0.04) |
| Chronic pulmonary disease | 0.14*** | (0.03) | -0.05 | (0.05) |
| Cerebrovascular disease | -0.02 | (0.06) | -0.08 | (0.07) |
| CHD or myocardial infarction | 0.13 | (0.06) | 0.11 | (0.09) |
| Dementia | -0.28** | (0.09) | -0.22 | (0.12) |
| Cancer | 0.32*** | (0.05) | 0.14* | (0.06) |
| Peptic ulcer disease | 0.14 | (0.07) | -0.17* | (0.06) |
| Peripheral vascular disease | -0.10 | (0.10) | 0.01 | (0.14) |
| Chronic kidney disease | 0.11* | (0.04) | -0.05 | (0.05) |
| Rheumatological disease | 0.19* | (0.07) | -0.04 | (0.07) |
| Liver disease | 0.47*** | (0.13) | 0.03 | (0.12) |
| Smoking *base: non-smoker* |  |  |  |  |
| Current or ex-smoker | 0.05 | (0.03) | -0.08* | (0.03) |
| **Practice characteristics** |  |  |  |  |
| Distance from practice to nearest general hospital *base 0-3km* |  |  |  |  |
| 3-6km | -0.07 | (0.04) | -0.08* | (0.04) |
| 6-9km | -0.12* | (0.06) | 0.07 | (0.04) |
| >9km | -0.03 | (0.06) | -0.01 | (0.06) |
| Distance from practice to nearest mental health inpatient facility *base 0-3km* |  |  |  |  |
| 3-6km | -0.09 | (0.05) | -0.09 | (0.05) |
| 6-9km | -0.16** | (0.05) | -0.10 | (0.05) |
| >9km | -0.19*** | (0.05) | -0.15* | (0.05) |
| N observations | 150,748 |  | 6,436 |  |
| N individuals | 16,485 |  | 3,596 |  |

Note: *p<0.05 **p<0.01 ***p<0.001. Standard errors adjusted for clustering at the practice level. Results from two part models: random effects probit and random effects glm second part (log link and gamma family) including quarter dummies and means of time-varying characteristics ((AR, CP, and, as the panel is unbalanced, quarter dummies)

**Supplementary Table 4.** **Regression results from two-part model for general hospital unplanned inpatient costs**

|  | Pr positive cost | | Cost if positive | |
| --- | --- | --- | --- | --- |
|  | β (se) | | β (se) | |
| **Primary care quality indicators** |  |  |  |  |
| Care plan | -0.07** | (0.02) | -0.03 | (0.03) |
| Annual review | -0.05* | (0.02) | 0.03 | (0.03) |
| **Patient characteristics** |  |  |  |  |
| Age at 1 April 2011 *base: 18-35 years* |  |  |  |  |
| 36-45 | -0.09** | (0.03) | 0.08 | (0.04) |
| 46-55 | -0.08* | (0.03) | 0.14** | (0.04) |
| 56-65 | -0.02 | (0.03) | 0.29*** | (0.05) |
| 66+ | 0.21*** | (0.04) | 0.37*** | (0.04) |
| Gender *base: female* |  |  |  |  |
| Male | -0.09*** | (0.02) | 0.04 | (0.03) |
| Ethnicity *base: Black and minority ethnicities* |  |  |  |  |
| White | 0.58*** | (0.03) | 0.01 | (0.04) |
| Index of Multiple Deprivation for area of residence *base: 1 (least disadvantaged)* |  |  |  |  |
| 2 | 0.03 | (0.04) | 0.06 | (0.04) |
| 3 | 0.11** | (0.04) | 0.09* | (0.04) |
| 4 | 0.15*** | (0.04) | 0.02 | (0.04) |
| 5 (most disadvantaged) | 0.19*** | (0.04) | 0.01 | (0.04) |
| Diagnosis category *base: bipolar* |  |  |  |  |
| Schizophrenia | 0.03 | (0.02) | 0.05 | (0.03) |
| Both diagnoses | 0.21*** | (0.03) | 0.09* | (0.03) |
| Number of years since diagnosis *base: 0-1* |  |  |  |  |
| 2-5 | -0.03 | (0.04) | 0.01 | (0.03) |
| 5+ | -0.12*** | (0.03) | 0.06 | (0.04) |
| Comorbidities *base: each not present* |  |  |  |  |
| Depression | 0.02 | (0.02) | -0.004 | (0.03) |
| Diabetes | 0.26*** | (0.04) | 0.13** | (0.04) |
| Chronic pulmonary disease | 0.14*** | (0.03) | -0.05 | (0.03) |
| Cerebrovascular disease | 0.12* | (0.06) | 0.004 | (0.05) |
| CHD or myocardial infarction | 0.29*** | (0.06) | 0.09 | (0.06) |
| Dementia | -0.01 | (0.08) | -0.16* | (0.07) |
| Cancer | 0.03 | (0.05) | 0.05 | (0.05) |
| Peptic ulcer disease | 0.14* | (0.06) | 0.05 | (0.07) |
| Peripheral vascular disease | 0.11 | (0.10) | 0.13 | (0.09) |
| Chronic kidney disease | 0.09* | (0.04) | 0.05 | (0.05) |
| Rheumatological disease | 0.10 | (0.07) | 0.04 | (0.07) |
| Liver disease | 0.40** | (0.13) | 0.15 | (0.12) |
| Smoking *base: non-smoker* |  |  |  |  |
| Current or ex-smoker | 0.03 | (0.03) | -0.01 | (0.03) |
| **Practice characteristics** |  |  |  |  |
| Distance from practice to nearest general hospital *base 0-3km* |  |  |  |  |
| 3-6km | -0.04 | (0.03) | -0.04 | (0.03) |
| 6-9km | -0.05 | (0.05) | 0.06 | (0.06) |
| >9km | -0.02 | (0.05) | -0.04 | (0.04) |
| Distance from practice to nearest mental health inpatient facility *base 0-3km* |  |  |  |  |
| 3-6km | -0.07* | (0.04) | -0.08* | (0.04) |
| 6-9km | -0.06 | (0.04) | -0.01 | (0.04) |
| >9km | -0.01 | (0.04) | -0.02 | (0.03) |
| N observations | 150,748 |  | 9,225 |  |
| N individuals | 16,485 |  | 4,713 |  |

Note: *p<0.05 **p<0.01 ***p<0.001. Standard errors adjusted for clustering at the practice level. Results from two part models: random effects probit and random effects glm second part (log link and gamma family) including quarter dummies and means of time-varying characteristics ((AR, CP, and, as the panel is unbalanced, quarter dummies)

**Supplementary Table 5.** **Regression results from two-part model for ED costs**

|  | Pr positive cost | | Cost if positive | |
| --- | --- | --- | --- | --- |
|  | β (se) | | β (se) | |
| **Primary care quality indicators** |  |  |  |  |
| Care plan | 0.02 | (0.02) | 0.00 | (0.02) |
| Annual review | -0.03* | (0.02) | 0.03 | (0.01) |
| **Patient characteristics** |  |  |  |  |
| Age at 1 April 2011 *base: 18-35 years* |  |  |  |  |
| 36-45 | -0.09*** | (0.03) | 0.02 | (0.02) |
| 46-55 | -0.17*** | (0.03) | 0.04* | (0.02) |
| 56-65 | -0.15*** | (0.03) | 0.06** | (0.02) |
| 66+ | -0.06* | (0.03) | 0.10*** | (0.02) |
| Gender *base: female* |  |  |  |  |
| Male | -0.04** | (0.02) | -0.01 | (0.01) |
| Ethnicity *base: Black and minority ethnicities* |  |  |  |  |
| White | 0.30*** | (0.02) | 0.10*** | (0.01) |
| Index of Multiple Deprivation for area of residence *base: 1 (least disadvantaged)* |  |  |  |  |
| 2 | 0.05 | (0.03) | 0.03 | (0.02) |
| 3 | 0.07* | (0.03) | 0.05** | (0.02) |
| 4 | 0.14*** | (0.03) | 0.06** | (0.02) |
| 5 (most disadvantaged) | 0.15*** | (0.03) | 0.05* | (0.02) |
| Diagnosis category *base: bipolar* |  |  |  |  |
| Schizophrenia | -0.03 | (0.02) | -0.00 | (0.01) |
| Both diagnoses | 0.01 | (0.02) | 0.03 | (0.02) |
| Number of years since diagnosis *base: 0-1* |  |  |  |  |
| 2-5 | -0.00 | (0.03) | 0.03 | (0.02) |
| 5+ | -0.09*** | (0.02) | 0.02 | (0.02) |
| Comorbidities *base: each not present* |  |  |  |  |
| Depression | 0.06*** | (0.02) | 0.00 | (0.01) |
| Diabetes | 0.20*** | (0.03) | 0.06** | (0.02) |
| Chronic pulmonary disease | 0.14*** | (0.02) | 0.03* | (0.01) |
| Cerebrovascular disease | 0.20*** | (0.04) | 0.03 | (0.02) |
| CHD or myocardial infarction | 0.17** | (0.05) | 0.06 | (0.03) |
| Dementia | 0.05 | (0.06) | 0.04 | (0.05) |
| Cancer | 0.05 | (0.04) | 0.00 | (0.02) |
| Peptic ulcer disease | 0.23*** | (0.05) | 0.08* | (0.03) |
| Peripheral vascular disease | 0.07 | (0.09) | 0.03 | (0.05) |
| Chronic kidney disease | 0.07* | (0.03) | 0.01 | (0.02) |
| Rheumatological disease | 0.17** | (0.06) | 0.02 | (0.03) |
| Liver disease | 0.34*** | (0.09) | 0.14* | (0.07) |
| Smoking *base: non-smoker* |  |  |  |  |
| Current or ex-smoker | 0.07*** | (0.02) | -0.01 | (0.01) |
| **Practice characteristics** |  |  |  |  |
| Distance from practice to nearest general hospital *base 0-3km* |  |  |  |  |
| 3-6km | -0.10*** | (0.02) | -0.01 | (0.02) |
| 6-9km | -.014*** | (0.04) | -0.03 | (0.02) |
| >9km | -0.10* | (0.03) | -0.00 | (0.02) |
| Distance from practice to nearest mental health inpatient facility *base 0-3km* |  |  |  |  |
| 3-6km | 0.03 | (0.03) | 0.01 | (0.03) |
| 6-9km | -0.03 | (0.04) | -0.00 | (0.02) |
| >9km | -0.07* | (0.03) | -0.04 | (0.02) |
| N observations | 150,748 |  | 13,883 |  |
| N individuals | 16,485 |  | 7,148 |  |

Note: *p<0.05 **p<0.01 ***p<0.001. Standard errors adjusted for clustering at the practice level. Results from two part models: random effects probit and random effects glm second part (log link and gamma family) including quarter dummies and means of time-varying characteristics ((AR, CP, and, as the panel is unbalanced, quarter dummies)

**Supplementary Table 6.** **Regression results from two-part model for specialist mental health costs**

|  | Pr positive cost | | Cost if positive | |
| --- | --- | --- | --- | --- |
|  | β (se) | | β (se) | |
| **Primary care quality indicators** |  |  |  |  |
| Care plan | 0.00 | (0.03) | -0.11*** | (0.02) |
| Annual review | -0.03 | (0.02) | -0.10*** | (0.02) |
| **Patient characteristics** |  |  |  |  |
| Age at 1 April 2011 *base: 18-35 years* |  |  |  |  |
| 36-45 | -0.14* | (0.06) | -0.15** | (0.05) |
| 46-55 | -0.30*** | (0.06) | -0.11* | (0.05) |
| 56-65 | -0.67*** | (0.07) | -0.18** | (0.06) |
| 66+ | -0.84*** | (0.07) | -0.31*** | (0.06) |
| Gender *base: female* |  |  |  |  |
| Male | 0.05 | (0.04) | -0.01 | (0.03) |
| Ethnicity *base: Black and minority ethnicities* |  |  |  |  |
| White | 0.69*** | (0.06) | 0.32*** | (0.04) |
| Index of Multiple Deprivation for area of residence *base: 1 (least disadvantaged)* |  |  |  |  |
| 2 | 0.11 | (0.08) | 0.02 | (0.06) |
| 3 | 0.16 | (0.09) | 0.02 | (0.07) |
| 4 | 0.36*** | (0.09) | -0.00 | (0.07) |
| 5 (most disadvantaged) | 0.57*** | (0.10) | 0.07 | (0.06) |
| Diagnosis category *base: bipolar* |  |  |  |  |
| Schizophrenia | 0.49*** | (0.05) | 0.23*** | (0.04) |
| Both diagnoses | 0.95*** | (0.08) | 0.53*** | (0.05) |
| Number of years since diagnosis *base: 0-1* |  |  |  |  |
| 2-5 | -0.47*** | (0.07) | 0.02 | (0.05) |
| 5+ | -0.68*** | (0.08) | 0.06 | (0.05) |
| Comorbidities *base: each not present* |  |  |  |  |
| Depression | -0.10* | (0.05) | -0.09** | (0.03) |
| Diabetes | 0.38*** | (0.07) | -0.01 | (0.05) |
| Chronic pulmonary disease | 0.05 | (0.05) | -0.01 | (0.03) |
| Cerebrovascular disease | -0.42** | (0.14) | -0.30** | (0.09) |
| CHD or myocardial infarction | -0.39** | (0.12) | 0.03 | (0.10) |
| Dementia | -0.25 | (0.15) | -0.40** | (0.13) |
| Cancer | -0.10 | (0.09) | -0.01 | (0.08) |
| Peptic ulcer disease | -0.04 | (0.13) | 0.02 | (0.10) |
| Peripheral vascular disease | -0.20 | (0.19) | -0.07 | (0.15) |
| Chronic kidney disease | -0.12 | (0.09) | -0.11 | (0.07) |
| Rheumatological disease | -0.25 | (0.15) | -0.05 | (0.11) |
| Liver disease | -0.24 | (0.26) | -0.27 | (0.17) |
| Smoking *base: non-smoker* |  |  |  |  |
| Current or ex-smoker | 0.11* | (0.05) | 0.02 | (0.04) |
| **Practice characteristics** |  |  |  |  |
| Distance from practice to nearest general hospital *base 0-3km* |  |  |  |  |
| 3-6km | -0.05 | (0.10) | -0.11 | (0.07) |
| 6-9km | -0.49** | (0.17) | 0.03 | (0.09) |
| >9km | 0.08 | (0.16) | -0.05 | (0.08) |
| Distance from practice to nearest mental health inpatient facility *base 0-3km* |  |  |  |  |
| 3-6km | 0.01 | (0.13) | -0.10 | (0.08) |
| 6-9km | 0.01 | (0.14) | 0.11 | (0.10) |
| >9km | -0.10 | (0.14) | -0.06 | (0.07) |
| N observations | 150,748 |  | 56,116 |  |
| N individuals | 16,485 |  | 9,732 |  |

Note: *p<0.05 **p<0.01 ***p<0.001. Standard errors adjusted for clustering at the practice level. Results from two part models: random effects probit and random effects glm second part (log link and gamma family) including quarter dummies and means of time-varying characteristics ((AR, CP, and, as the panel is unbalanced, quarter dummies)
